# Supplementary material for: Helicobacter pylori in Native Americans in Northern Arizona
Source: Diseases. 2022 Mar 23;10(2):19. doi: 10.3390/diseases10020019 (PMC9036257; doi:10.3390/diseases10020019)
Supplement: Supplementary file 1 [file diseases-10-00019-s001.zip › diseases-1636941-supplementary.pdf]

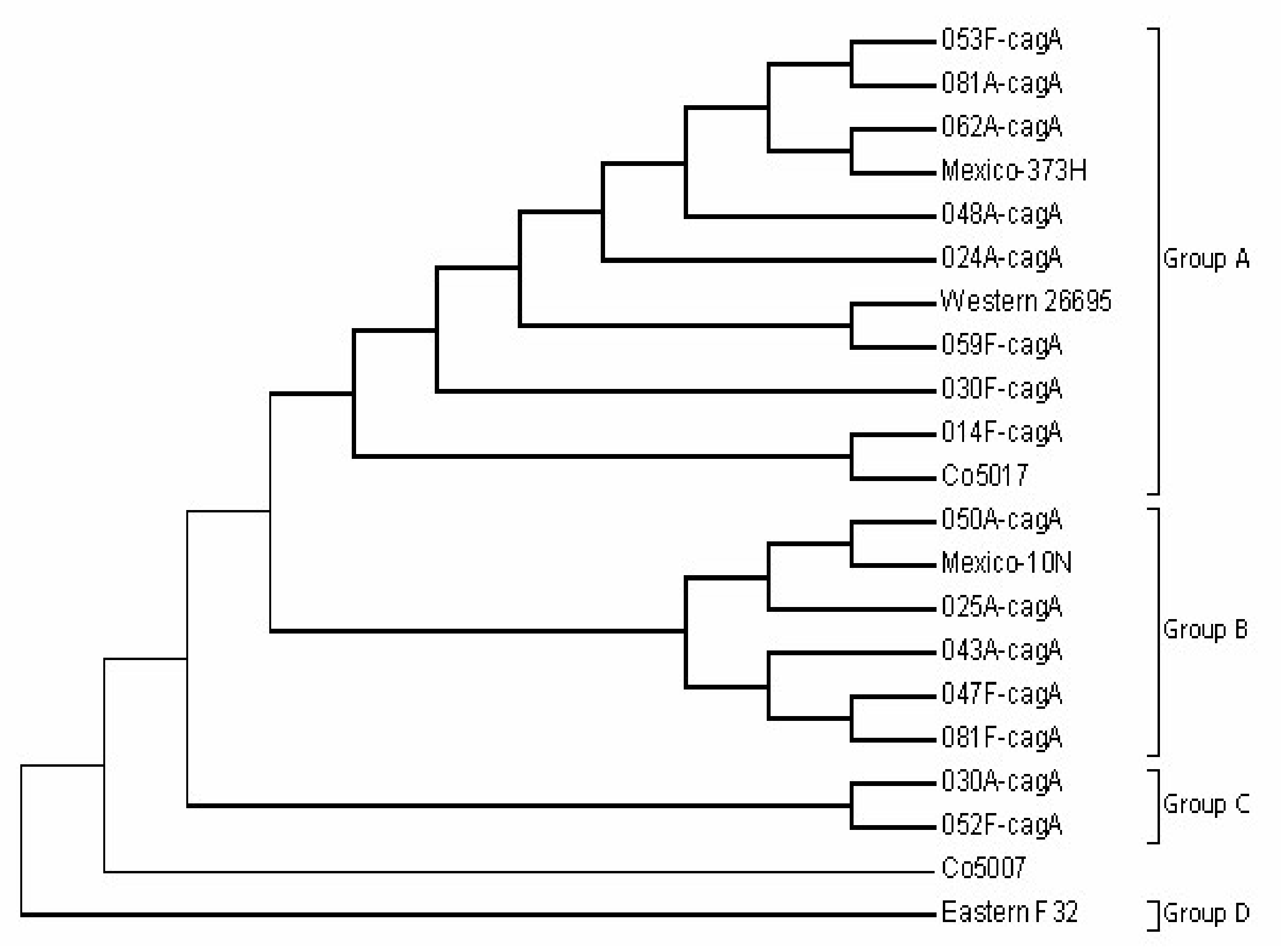

Figure S1. Phylogenetic tree of *cagA* in Navajo isolates compared with isolates from Mexico, 373H (JN390445.1) and 10N (JN390446.1); Colombia, Co5007 (EU251000.1) and Co5017 (EU250993.1); a Western (NP207343.1); and an Eastern *H. pylori* strain isolates (AAF17597.1).

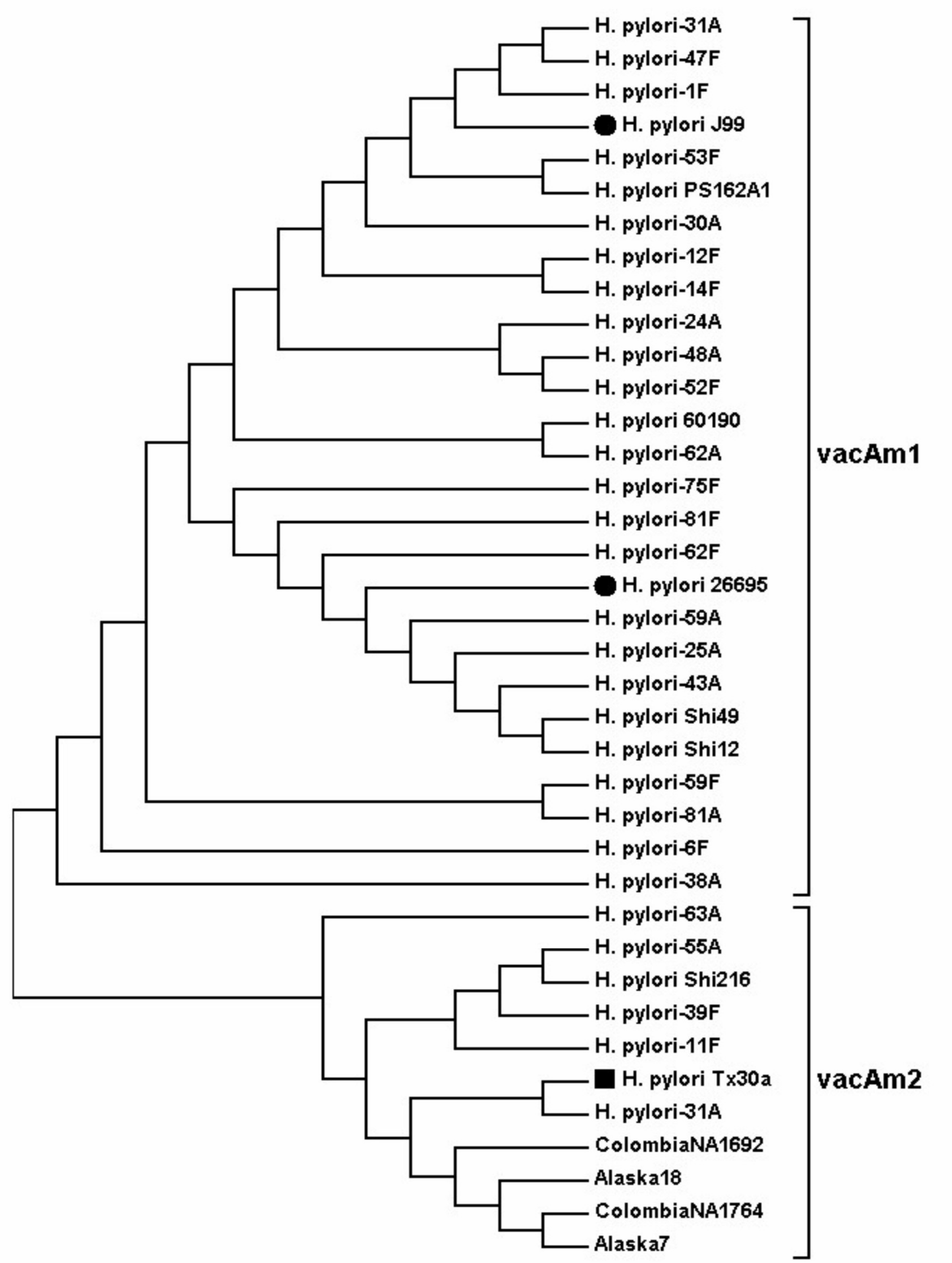

Figure S2. Phylogenetic tree of Navajo *vacA*m alleles in *Helicobacter pylori* sequences. *vacA*m1 sequences are on top with two typical m1 reference strains (USA2754 - AB057223.1, 60190 - U05676.1, and J99 – NC 000921.1). The *vacA*m2 sequences are below and contain the reference strain Tx30a (U29401.1), as well as Colombian (Co1692 – AB057311.1, Co1764 – A057313.1, PS162A1 – GU06444.1), Alaskan (Alaska7 – AB057294.1, Alaska 18 – AB057305.1), and Peruvian Amazon (Shi49 – GU064499.1, Shi12 – GU064505.1, Shi216 – GU064505.1) isolates.
